# Mie-Metamaterials-Based Thermal Emitter for Near-Field Thermophotovoltaic Systems

**DOI:** 10.3390/ma10080885

**Published:** 2017-07-31

**Authors:** Alok Ghanekar, Yanpei Tian, Sinong Zhang, Yali Cui, Yi Zheng

**Affiliations:** 1 Department of Mechanical, Industrial and Systems Engineering, University of Rhode Island, Kingston, RI 02881, USA; alokg@my.uri.edu (A.G.); yanpei_tian@my.uri.edu (Y.T.); 2 College of Life Sciences, Northwest University, Xi’an 710069, China; zhangsinong@stumail.nwu.edu.cn (S.Z.); yalicui@nwu.edu.cn (Y.C.); 3 National Engineering Research Center for Miniaturized Detection Systems, Northwest University, Xi’an 710069, China

**Keywords:** near-field thermal radiation, thermophotovoltaics, Mie-metamaterials, effective medium theory

## Abstract

In this work, we theoretically analyze the performance characteristics of a near-field thermophotovoltaic system consisting a Mie-metamaterial emitter and GaSb-based photovoltaic cell at separations less than the thermal wavelength. The emitter consists of a tungsten nanoparticle-embedded thin film of SiO${}_{2}$ deposited on bulk tungsten. Numerical results presented here are obtained using formulae derived from dyadic Green’s function formalism and Maxwell-Garnett-Mie theory. We show that via the inclusion of tungsten nanoparticles, the thin layer of SiO${}_{2}$ acts like an effective medium that enhances selective radiative heat transfer for the photons above the band gap of GaSb. We analyze thermophotovoltaic (TPV) performance for various volume fractions of tungsten nanoparticles and thicknesses of SiO${}_{2}$.

## 1. Introduction

Thermophotovoltaics (TPVs) have been the focus of several works as an alternative to power generation technologies and a technology for waste heat recovery systems [1,2,3]. A typical TPV system consists of a high-temperature (∼1500 K) thermal emitter and a photovoltaic (PV) cell that converts the energy of incident photons into electricity. While ideal TPV systems convert radiative energy into electricity at an efficiency of the Carnot engine, practical TPV systems suffer from mismatch between the emission spectra of the emitter and absorption spectra of PV cell [4,5,6,7,8]. Several studies have investigated the use of metamaterials [9,10,11], surface gratings and photonics crystals [12,13,14,15,16,17], and complex surface patterns [18] to improve efficiency of TPV systems. In the last few years, it was demonstrated that near-field thermal radiation has a great potential in improving TPV systems [19,20,21,22,23,24]. Several theoretical studies were focused on exploiting near-field coupling of surface waves between emitter and PV cell at nanometer separation to improve the efficiency. It is challenging to develop materials that can withstand high temperatures as well as allow an enhanced coupling of surface waves for the energies above the band gap of the PV cell. One-dimensional gratings, hyperbolic metamaterials, and photonic crystals have shown a great potential in TPV applications. Dielectric mixtures and nanoparticle-embedded thin films can also be utilized to tune near-field thermal radiation [25]. It has been previously demonstrated that Mie-metamaterials or Mie-resonance metamaterials that utilize Mie resonances of inclusions into host materials can be used for spectral tuning of near-field thermal radiation. Authors demonstrated the possible use of tungsten nanoparticle-embedded thin film of SiO${}_{2}$ to achieve a selective thermal emitter for a far-field TPV system [26]. The effect of nanoparticle inclusions into host material on near-field radiative heat transfer has been investigated in several theoretical studies [27,28,29]. Stemming from earlier work, we demonstrate the use of a Mie-metamaterial thermal emitter that consists of tungsten nanoparticles embedded in a thin film of SiO${}_{2}$ deposited on a thick layer of tungsten for a near-field TPV system. We also explore possible alternative of W nanoparticles by refractive materials such as titanium nitride (TiN), tantalum (Ta), and molybdenum (Mo). We investigate the performance of such a TPV system for various configurations. While many recent works have dealt with gratings, photonic crystals, and other metamaterials, this is the first time nanoparticle-embedded thin films have been investigated for a near-field TPV system.

**Figure 1:**
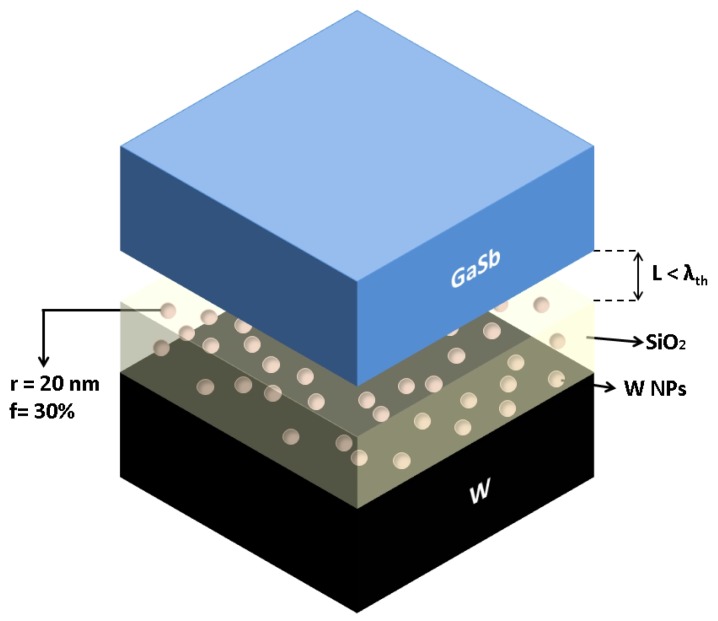
Schematic of near-field thermophotovoltaic system consisting of the proposed thermal emitter and GaSb-based PV cell at separation less than the thermal wavelength.

The configuration of the near-field TPV system considered in the present study is shown in Figure 1. The emitter side is a Mie-metamaterial consisting of tungsten nanoparticles embedded in SiO${}_{2}$ thin film on the top of a tungsten layer. The radius of the nanoparticles was fixed at 20 nm. The volume fraction and thickness of the SiO${}_{2}$ layer can be varied to investigate behaviour of the system. The layer of tungsten blocks radiation from the substrate, making the emitter essentially opaque. The PV cell considered here is GaSb (which has a bandgap of 0.726 eV), whose properties can be found in Reference [30]. Calculations reported in this work are for emitter temperature of 1500 K, while the PV cell is assumed to be at 300 K. The separation between the emitter and PV cell is comparable to or less than the thermal wavelength at 1500 K ($\lambda_{th}$= 1.93 μm).

While we mostly concern ourselves with the theoretical side of the proposed thermal emitter, there are various ways to fabricate nanoparticle-embedded thin films. For example, it has been demonstrated that a stack of metallic nanoparticle arrays and SiO${}_{2}$ arrays can be fabricated [31]. Alternatively, as suggested by [32], core-shell nanoparticle arrays can be fabricated using tungsten core and SiO${}_{2}$ shell. This is followed by sputtering or chemical vapor deposition of SiO${}_{2}$. W Nanoparticles-SiO${}_{2}$ composites can also be fabricated using sputtering deposition process [33,34,35]. A mixture of W and SiO${}_{2}$ powders can be prepared using PVDF (polyvinylidene fluoride) and sintering/pressing onto a sputtering cooling plate. W-SiO${}_{2}$ composites can be fabricated by RF sputtering of W followed by sputtering of W-SiO${}_{2}$ powder. During the sputtering process, tungsten nanoparticles would form.

## 2. Results and Discussion

**Figure 2:**
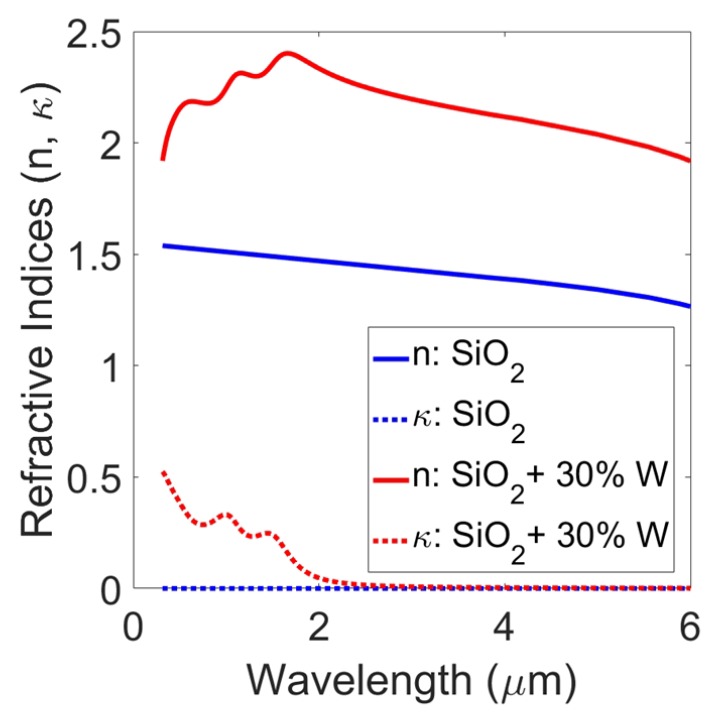
Real (n) and imaginary (κ) parts of refractive indices of pure SiO${}_{2}$ and SiO${}_{2}$ with 30% tungsten nanoparticles.

The refractive indices of plain SiO${}_{2}$ and that of SiO${}_{2}$ mixed with 30% nanoparticles of tungsten are shown in Figure 2. The effect of nanoparticle inclusions can be observed. SiO${}_{2}$ has a near-constant value of refractive index (*n*) and a negligible extinction coefficient (κ) in the spectral range of our interest. The dielectric mixture of SiO${}_{2}$ and tungsten displayed an overall increased effective refractive index and a higher absorption coefficient for wavelengths shorter than 2.5 μm.

**Figure 3:**
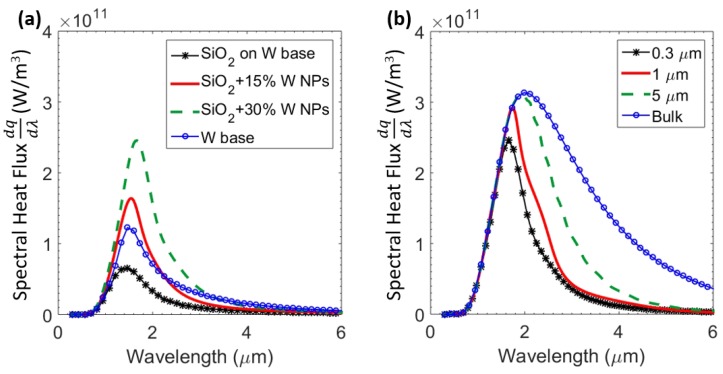
Spectral heat flux across the proposed emitter and the GaSb photovoltaic (PV) cell at a separation of L = 100 nm for (**a**) various volume fractions of W nanoparticles—0%, 15% and 30%—compared to bulk W emitter; (**b**) Various thicknesses of SiO${}_{2}$ layer—0.3 μm, 1 μm, 5 μm, and bulk respectively.

It is crucial to reduce the spectral energy below the band-gap of the PV cell in order to improve the overall thermal efficiency of the TPV system. The coupling of surface waves dictates whether radiative transfer would be enhanced or suppressed. The goal is to enhance radiative transfer above the bandgap without significantly enhancing radiation below the bandgap of the PV cell. In order to assess the impact of nanoparticle inclusions, we investigate the spectral heat flux of the proposed TPV system for various configurations. The spectral heat flux across the proposed thermal emitter at a separation of L=100 nm for various compositions is plotted in Figure 3. The emitter with a 0.3 μm layer of SiO${}_{2}$ on tungsten had lower heat flux across the spectrum when compared to plain tungsten, as seen in Figure 3a. However, upon the inclusion of tungsten nanoparticles, spectral heat flux increased and was more selective towards shorter wavelengths (λ<2 μm). While spectral heat flux showed an increase over the entire range, it was more prominent for energies above the band-gap, leading to a lesser fraction of energy lost. An increased absorption coefficient in the shorter wavelengths can be accounted for by enhanced coupling of surface modes in that range. To demonstrate the effect of reducing bulk layer to thin layer, Figure 3b illustrates spectral responses for various thicknesses of SiO${}_{2}$ layer and a fixed nanoparticle volume fraction of 30%. While a bulk layer of SiO${}_{2}$ mixed with tungsten nanoparticle showed broadband heat transfer, it can be clearly seen that thinner layers of SiO${}_{2}$ yielded more selective spectral response while maximum spectral heat flux remained relatively constant. It can be observed that radiation at longer wavelengths was more sensitive to layer thickness—such a configuration is more desirable for minimizing losses due to long-wavelength photons. In principle, it is possible to tune the near-field thermal radiation by changing the volume fraction of nanoparticles and thickness of the SiO${}_{2}$ layer to achieve optimal configuration for a given operating temperature and separation.

**Figure 4:**
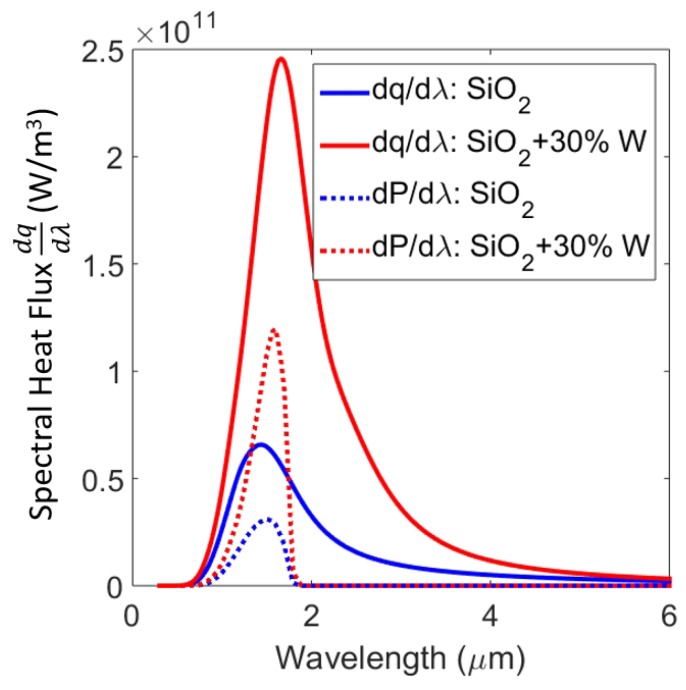
Predicted spectral density of output power (dashed lines) from GaSb PV cell for emitter with pure SiO${}_{2}$ thin film and SiO${}_{2}$ with 30% of W nanoparticles for a separation of 100 nm compared with corresponding spectral heat fluxes (solid lines).

**Figure 5:**
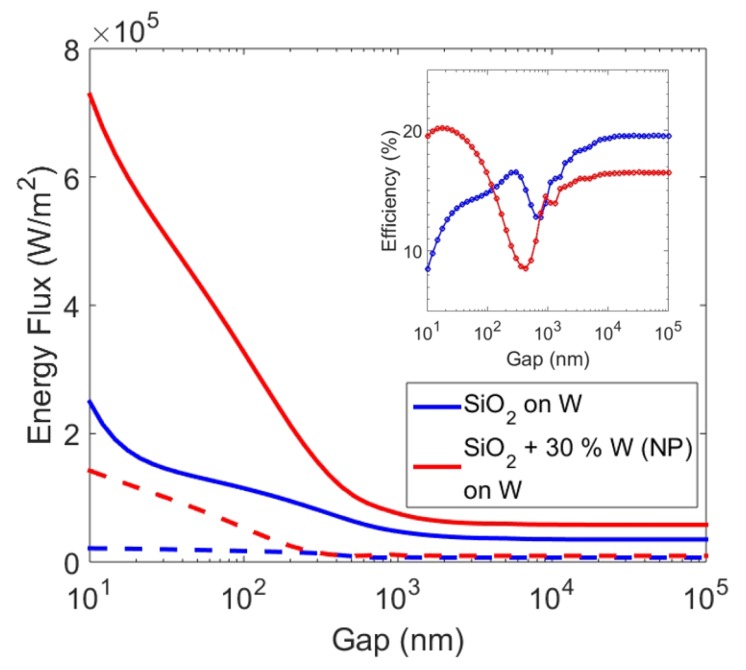
Total heat flux (solid lines) and output power (dashed lines) of PV cell as a function of separation between the emitter and PV cell for an emitter of pure SiO${}_{2}$ film of 0.5 μm and SiO${}_{2}$ films with W nanoparticles. Inset shows overall efficiency of the corresponding thermophotovoltaic (TPV) systems plotted as a function of separation. NP: nanoparticle.

**Figure 6:**
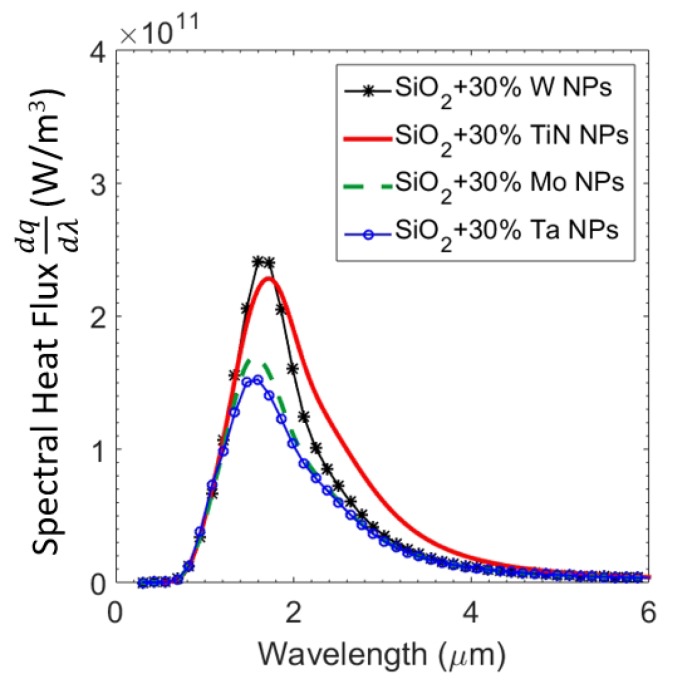
Spectral heat flux across the emitter consisting of nanoparticles of alternative materials and the GaSb PV cell at a separation of L=100 nm for a nanoparticles volume fraction of 30%.

We now investigate the power output of the GaSb PV cell as a result of near-field radiative heat transfer at a separation of 100 nm. To assess the overall performance of the TPV system, we model the PV cell as discussed in Section 3. We calculate spectral density of output power along with the total output power. Figure 4 shows calculated spectral density of output power from the PV cell for the emitter with a pure SiO${}_{2}$ layer of 0.3 μm thickness on tungsten and a SiO${}_{2}$ layer with 30% tungsten nanoparticles. The emitter with a tungsten nanoparticles-embedded thin film of SiO${}_{2}$ displayed an enhanced output power contribution above the bandgap of the GaSb PV cell when compared with the emitter with pure SiO${}_{2}$ layer. For comparison, the spectral heat flux across the interface of the same configurations are also shown. The total heat flux and total power output of the PV cell of these configurations are plotted against separation up to 10 nm in Figure 5. For gaps larger than 1 μm (far-field), heat flux (and consequently power power and system efficiencies) were independent of the distance. For separations less than 1 μm (near-field), the overall heat flux increased due to the presence of evanescent waves. Consequently, the output power also rose monotonically as separation between the emitter and PV cell was reduced. The inset in Figure 5 shows TPV system efficiency against distance for the same setups. While the trend in efficiencies is not as monotonous, for separations smaller than 100 nm, the emitter with tungsten nanoparticles showed higher thermal efficiency than the one with pure SiO${}_{2}$. This is supported by earlier results that show the increased selectivity at shorter wavelengths leading to lower losses. Interestingly, the emitter with pure SiO${}_{2}$ had higher efficiency in the far-field. Nevertheless, such a configuration had lower output power. The oscillatory behaviour of efficiency has been observed before, and can be attributed to vacuum gap behaving like a waveguide [4]. We would like to emphasize that in order to further improve the performance of the TPV system, the materials chosen are not necessarily optimal. For example, PV cells with lower band gap (e.g., InGaAs and InGaSb or quaternary alloys like InGaAsSb) can be used. This can allow the use of lower emitter temperature or higher efficiencies and output power at the same operating temperatures. Alternatively, other materials, such as Al${}_{2}$O${}_{3}$ (in place of SiO${}_{2}$) and platinum, molybdenum, tantalum, and titanium nitride (in place of W) can be investigated for thermal emitter structure. We show a possible replacement of W by refractory materials such as titanium nitride (TiN), molybdenum (Mo), and tantalum (Ta) using our calculations in Figure 6. Spectral heat flux between the PV cell and emitter with SiO${}_{2}$ layer of 0.3 μm thickness and 30% nanoparticles at a separation of 100 nm is shown. For comparison, heat flux with tungsten nanoparticles is shown. Corresponding values of total heat flux for W, TiN, Mo, and Ta nanoparticles were 3.72 × 10${}^{5}$, 3.81 × 10${}^{5}$, 2.68 × 10${}^{5}$ and 2.57 × 10${}^{5}$ W/m${}^{2}$, respectively. From Figure 6 it appears that emitter with TiN and W nanoparticles displayed higher heat flux for this particular configuration. The emitter with tungsten nanoparticles showed better selectivity than TiN. Various combinations of material type and dimensions can be investigated to tune the emission spectra.

Overall, we have conducted the first numerical investigation of a near-field thermophotovoltaic system that uses a Mie-metamaterial-based thermal emitter and a PV cell at a separation less than the thermal wavelength. We have theoretically demonstrated an enhanced wavelength-selective thermal emitter for near-field thermophotovoltaic system using a Mie-resonance metamaterial. The thermal emitter consists of tungsten nanoparticle-embedded thin film of SiO${}_{2}$ deposited on a thick tungsten substrate. We analyzed the performance of such a TPV device for various cases. We studied the effect of volume fraction, layer thickness of SiO${}_{2}$, and separation between emitter and PV cell. The embedded tungsten nanoparticles in the thin film can alter the refractive index of the film and allow spectral control of near-field radiative transfer across the emitter and the PV cell. We evaluated the energy conversion efficiency of the proposed near-field thermophotovoltaic system. The results show that the structure of the Mie-metamaterial thermal emitter can significantly improve the efficiency of the thermophotovoltaic system. Improvement in spectral selectivity as well as overall heat transfer can be attributed to increased power output and efficiency. We showed that by changing the volume fraction of nanoparticles and the thickness of the SiO${}_{2}$ layer, it is possible to tune the near-field thermal radiation to obtain enhanced output power and high thermal efficiency. The materials considered can withstand high temperatures and are suitable for thermal emitters.

## 3. Materials and Methods

The expression of radiative transfer between closely-spaced bodies can be derived using dyadic Green’s function approach [36], and is given by

(1) $$q_{1\to 2}\left(T_{1},\;T_{2},\;L\right)\;=\;\;\int_{0}^{\infty }\;\frac{d\omega }{2\pi }\left[\Theta \left(\omega ,T_{1}\right)\;-\;\Theta \left(\omega ,T_{2}\right)\right]T_{1\to 2}\left(\omega \right)\;,$$

where $\Theta \left(\omega ,T\right)=\left(\hbar \omega /2\right)\coth\left(\hbar \omega /2k_{B}T\right)$ is the energy of a harmonic oscillator at frequency ω, temperature *T*, *ћ* is the reduced Planck constant, and $k_{B}$ is the Boltzmann constant. The function $T_{1\to 2}\left(\omega \right)$ corresponds to the spectral transmissivity in radiative transfer between media 1 and 2 separated by distance *L* and is expressed as [36]

(2) $$\begin{array}{cc} \;\;\;T_{1\to 2}\left(\omega \right)\;=\; & \int_{0}^{\omega /c}\;\frac{k_{\rho }dk_{\rho }}{2\pi }\;\;\sum_{\mu =s,p}\;\frac{\left(1-\right|{\tilde{R}}_{h1}^{\left(\mu \right)}{|}^{2})(1-|{\tilde{R}}_{h2}^{\left(\mu \right)}{|}^{2})}{|1-{\tilde{R}}_{h1}^{\left(\mu \right)}{\tilde{R}}_{h2}^{\left(\mu \right)}e^{2jk_{hz}L}{|}^{2}}\\ + & \int_{\omega /c}^{\infty }\;\frac{k_{\rho }dk_{\rho }}{2\pi }\;\;\sum_{\mu =s,p}\;\frac{4ℑ\left({\tilde{R}}_{h1}^{\left(\mu \right)}\right)ℑ\left({\tilde{R}}_{h2}^{\left(\mu \right)}\right)e^{-2|k_{hz}|L}}{|1-{\tilde{R}}_{h1}^{\left(\mu \right)}{\tilde{R}}_{h2}^{\left(\mu \right)}e^{-2|k_{hz}|L}{|}^{2}}\;, \end{array}$$

where ${\tilde{R}}_{h1}^{\left(\mu \right)}$ and ${\tilde{R}}_{h2}^{\left(\mu \right)}$ are polarized effective reflection coefficients of the two half spaces (calculated in the absence of other half space), and $k_{hz}$ is the *z*-component of wavevector in vacuum. The first term in Equation (2) corresponds to propagating waves, while the second term describes the thermal transport due to evanescent waves, and its contribution is significant only for small values of gap *L*. For a structure having *N*-layer media having (N-1) interfaces, the expression for the generalized reflection coefficient at the interface between region *i* and region i+1 is given by [37]:

(3) $${\tilde{R}}_{i,i+1}^{\left(\mu \right)}=\frac{R_{i,i+1}^{\left(\mu \right)}+{\tilde{R}}_{i+1,i+2}^{\left(\mu \right)}e^{2jk_{i+1,z}\left(d_{i+1}-d_{i}\right)}}{1+R_{i,i+1}^{\left(\mu \right)}{\tilde{R}}_{i+1,i+2}^{\left(\mu \right)}e^{2jk_{i+1,z}\left(d_{i+1}-d_{i}\right)}}\;,$$

where $R_{i,i+1}^{\left(\mu \right)}$ is the Fresnel reflection coefficient at the interface between layers *i* and i+1, and ${\tilde{R}}_{i+1,i+2}^{\left(\mu \right)}$ is the generalized reflection coefficient at the interface between layers i+1 and i+2, μ=s (or *p*) refers to transverse electric (or magnetic) polarization, $z=-d_{i}$ is the location of the *i*th interface. $k_{i,z}=\sqrt{\varepsilon_{i}\left(\omega \right)\omega^{2}/c^{2}-k_{\rho }^{2}}$ is the normal *z*-component of the wave vector in medium *i*, wherein $\varepsilon_{i}\left(\omega \right)$ is the relative permittivity of the medium *i* as a function of angular frequency ω, *c* is the speed of light in vacuum, and $k_{\rho }$ is the magnitude of the in-plane wave vector. With ${\tilde{R}}_{N,N+1}^{\left(\mu \right)}=0$, the above equation provides a recursive relation to calculate the reflection coefficients ${\tilde{R}}_{i,i+1}^{\left(\mu \right)}$ in all regions. To calculate the effective dielectric function of the Mie-metamaterial, we use the Clausius–Mossotti equation [38,39].

(4) $$\varepsilon_{eff}=\varepsilon_{m}\left(\frac{r^{3}+2\alpha_{r}f}{r^{3}-\alpha_{r}f}\right)\;,$$

where $\varepsilon_{m}$ is the dielectric function of the matrix, $\alpha_{r}$ is the electric dipole polarizability, and *r* and *f* are the radius and volume fraction of nanoparticles, respectively. To consider the size effects of nanoparticle inclusions, we use the Maxwell-Garnett formula, which employs the expression for electric dipole polarizability using Mie theory [40], $\alpha_{r}=3jc^{3}a_{1,r}/2\omega^{3}\varepsilon_{m}^{3/2}$, where $a_{1,r}$ is the first electric Mie coefficient given by

(5) $$a_{1,r}\;=\;\frac{\sqrt{\varepsilon_{np}}\psi_{1}\left(x_{np}\right)\psi_{1}^{{}^{\prime }}\left(x_{m}\right)\;-\;\sqrt{\varepsilon_{m}}\psi_{1}\left(x_{m}\right)\psi_{1}^{{}^{\prime }}\left(x_{np}\right)}{\sqrt{\varepsilon_{np}}\psi_{1}\left(x_{np}\right)\xi_{1}^{{}^{\prime }}\left(x_{m}\right)\;-\;\sqrt{\varepsilon_{m}}\xi_{1}\left(x_{m}\right)\psi_{1}^{{}^{\prime }}\left(x_{np}\right)}\;,$$

where $\psi_{1}$ and $\xi_{1}$ are Riccati–Bessel functions of the first order given by $\psi_{1}\left(x\right)=xj_{1}\left(x\right)$ and $\xi_{1}\left(x\right)=xh_{1}^{\left(1\right)}\left(x\right)$ where $j_{1}$ and $h_{1}^{\left(1\right)}$ are first-order spherical Bessel functions and spherical Hankel functions of the first kind, respectively. Here, “${\;}^{\prime }\;$” indicates the first derivative. $x_{m}=\omega r\sqrt{\varepsilon_{m}}/c$ and $x_{np}=\omega r\sqrt{\varepsilon_{np}}/c$ are the size parameters of the matrix and the nanoparticles, respectively; $\varepsilon_{np}$ is the dielectric function of nanoparticles. It is worth mentioning that Maxwell-Garnett-Mie theory is applicable when the average distance between inclusions is much smaller than the wavelength of interest [41]. This criteria is satisfied in the calculations presented. Since nanoparticle diameter (40 nm) is much smaller than the thickness of the thin film (0.3 μm) considered, effective medium theory holds true for the calculations presented in this study. Dielectric functions of the materials (SiO${}_{2}$ and W) considered in this paper are taken from literature [42,43]. Having very low temperature coefficients, room temperature values of dielectric function are used for SiO${}_{2}$ [44]. Dielectric properties of tungsten were also assumed to be unchanged, as the operating temperature is much less than the melting point.

Near-field thermal radiation and charge transport in PV cell can be theoretically modelled by accounting for charge density distribution due to the number of photons absorbed at different cell depths [22]. Due to the limitations of the present study, we employ a simplistic model to calculate the output power of a PV cell. We assume that quantum efficiency of a PV cell in the near-field would be the same as that in the far-field. Therefore, short circuit current can be calculated as [22]

(6) $$I_{SC}=\int_{E_{g}/\hbar }^{\infty }\frac{e}{\hbar \omega }\cdot EQE_{GaSb}\left(\omega \right)\cdot \frac{dq}{d\omega }d\omega \;,$$

where $E_{g}$ is the bandgap of GaSb cell, $EQE_{GaSb}$ is external quantum efficiency, dq/dω is spectral heat flux, and *e* is the electronic charge. Dark current can be calculated by

(7) $$I_{0}=\frac{eD_{n}n_{i}^{2}}{L_{n}N_{n}}+\frac{eD_{p}n_{i}^{2}}{L_{p}N_{p}},$$

where $D_{n}$ and $D_{p}$ are diffusivities of electrons and holes, respectively, $n_{i}$ is the intrinsic carrier concentration, $N_{n}$ and $N_{p}$ are concentrations of electrons and holes, respectively, while diffusion lengths $L_{n}$ and $L_{p}$ can be calculated in terms of diffusivity and recombination lifetime τ using

(8) $$L_{x}=\sqrt{D\cdot \tau }\;.$$

Total recombination lifetime is calculated by

(9) $$1/t_{tot}=1/\tau_{R}+1/\tau_{SHR}+1/\tau_{Au}\;,$$

where $\tau_{R}$, $\tau_{SHR}$, and $\tau_{Au}$ are radiative recombination, Shockley–Hall recombination, and Augar recombination, respectively [45]. Open circuit voltage is calculated using

(10) $$V_{OC}=\left(k_{B}T_{PV}/e\right)ln\left(I_{SC}/I_{0}+1\right)\;.$$

Output power of the PV cell is given by

(11) $$P_{out}=I_{SC}V_{OC}\left(1-1/z\right)\left[1-ln\left(z\right)/z\right]\;,$$

where $z=ln(I_{SC}/I_{0})$ and efficiency of the TPV system is given by

(12) $$\eta =P_{out}/Q_{rad}\;.$$

For our calculations, intrinsic carrier concentration was assumed to be $4.3\times 10^{12}$ cm${}^{-3}$. Carrier concentration of electrons and holes were assumed to be equal to $N_{n}=N_{p}=10^{17}$ cm${}^{-3}$. The recombination lifetimes are taken to be $\tau_{R}=40$ ns, $\tau_{SHR}=10$ ns, and $\tau_{Au}=20$ μs. Carrier diffusivities are $D_{n}=129$ cm${}^{2}/$s and $D_{p}=39$ cm${}^{2}$/s for electrons and holes, respectively.
